# Acknowledgment of *Ad Hoc* Reviewers

**DOI:** 10.1128/mSphere.01235-20

**Published:** 2020-12-23

**Authors:** Michael J. Imperiale

**Affiliations:** a University of Michigan Medical School, Ann Arbor, Michigan, USA

## EDITORIAL

Each year at this time, it has been my tradition to provide a brief summary of our progress and to thank those of you who have reviewed manuscripts during the year for your service to *mSphere*, the American Society for Microbiology, and the scientific enterprise overall. The COVID-19 pandemic has caused us to operate differently with respect to logistics, but fortunately our reviewers have been willing and able to help our authors by providing fair, thoughtful reviews. For doing this while dealing with all the stresses that the pandemic has rained upon us, this year you have my extra special gratitude and that of our senior editors and editors.

Despite the pandemic, our submission numbers have greatly increased (>1,200 as of Thanksgiving). Our team interprets this as meaning that you, our authors, have trust in our process and the quality that the name *mSphere* carries with it. I therefore thank all of you who have submitted manuscripts in 2020 for that vote of confidence.

At the beginning of the pandemic, I offered some tips that I thought would help us support each other and the public whom we serve as scientists (1). It is my sense that we, the microbial sciences community, have stepped up to the challenge. Many of us pivoted our research and development programs and our clinical microbiology labs to contribute to the global response to the virus. Our previous foundational work on coronaviruses and other pathogens, on the immune response, and on drug and vaccine development positioned the world to be able to respond to this pandemic in a remarkable manner. I have seen the way in which we have helped each other during these stressful times and am encouraged by our resilience.

As the end of the year approaches, I hope that you all have a chance to take some time off to enjoy with your family and friends (even if it is in a virtual setting).

Again, my sincere thanks to all of you for being part of the *mSphere* family. Let’s look forward together to 2021.

Peter Aaby
Zachary Aanderud
Amr Mahmoud Abd El-Gawad
Akio Abe
Lisa Abernathy-Close
Wolf-Rainer Abraham
Mark Achtman
Felise G. Adams
Mark D. Adams
Joshua N. Adkins
Philippe V. Afonso
Ameeta K. Agarwal
Alain Bernardin Agnememel
Hector C. Aguilar
Panblo Aguilar
Nacho Aguilo
Danielle Ahn
Shimpei Aikawa
Vishukumar Aimanianda
Gillian M. Air
Matthew Akers
Mustafa Akkoyunlu
Fadhl Alakwaa
Ashfaqul Alam
Ana Alastruey-Izquierdo
John F. Alcorn
Howbeer Muhamad Ali
Alexandre Almeida
Uri Alon
Francis Alonzo
David Alsteens
Carmen Amaro
Jorge Amich
Karthik Anantharaman
Cheryl P. Andam
Christopher Anderson
Matthew Zack Anderson
Dan I. Andersson
Raul Andino
Diego O. Andrey
Elliot J. Androphy
Esther R. Angert
Juan Anguita
Vijay C. Antharam
Alberto Antonelli
Joseph Antony
Yoshichika Arakawa
Zachary Ardern
Angela Arenas-Gamboa
Silvia Argimon
José M. Argüello
David M. Aronoff
Jyoti Arora
Gustavo Arrizabalaga
Michel Arthur
Sassan Asgari
Ina Atrée
Ahmed Sherif Attia
Walter J. Atwood
Jennifer M. Auchtung
Sarit Avrani
Gordon A. Awandare
M. Andrea Azcarate-Peril
Brian D. Badgley
Victor M. Baizabal-Aguirre
Lauren O. Bakaletz
Steven Baker
Susan C. Baker
Megan Tierney Baldridge
Cynthia Baldwin
Carl J. Balibar
Jimmy D. Ballard
Elizabeth Ballou
Ludmila Baltazar
David A. Baltrus
Robert Bambara
Connor Gavin George Bamford
Gert Bange
James D. Bangs
Edel Figueiredo Barbosa-Stancioli
Antonio Barragan
Christopher F. Basler
Christine Marie Bassis
Philippe Bastin
Florian F. Bauer
Sal Baxamusa
Navid Bazghaleh
Bernard W. Beall
Marco Becherelli
Josh R. Beck
Stephan Becker
Sebastian Behrens
Deborah Bell-Pedersen
George A. Belov
Sarah Ben Maamar
Richard J. Bennett
Teresa M. Bergholz
Ales Berlec
Brent Berwin
Charles L. Bevins
Sinem Beyhan
Amin Talebi Bezmin Abadi
Purnima Bhanot
Jordan E. Bisanz
S. Bishajit
Raphael Bisinotto
Jessica Mary Alice Blair
Steven R. Blanke
Mark R. Bleackley
Regina B. Bledsoe
Bradley J. Blitvich
Patricia Pringle Bloom
Antje Blumenthal
Kasun H. Bodawatta
Jason M. Bodily
Patrick Boerlin
Gregory Bokinsky
Nicholas Andrew Bokulich
Adrianus C. Boon
Ray Borrow
Valeria Bortolaia
Thomas C. G. Bosch
Anna Both
Elsa N. Bou Ghanem
Philippe Bouloc
Sebastien Boutin
Anna Bowman
Eric Boyd
Ethna Fidelma Boyd
Tess Elizabeth Brewer
Shaun R. Brinsmade
Jeremy S. Brown
Jessica C. S. Brown
Michael G. Brown
John H. Brumell
James Brust
Arielle M. Bryan
Wolfgang Buckel
Michelle Monique Conni Buckner
Carlos Bueno
Tucker Burch
Lindsey R. Burcham
Cara C. Burns
Philip Brandon Busbee
Sarah N. Buss
Mark J. Buttner
Jerry M. Buysse
Mariana X. Byndloss
Lauren Byrd-Leotis
David J. Bzik
Matthew Cabeen
Ken Cadwell
Laty A. Cahoon
Richard A. Calderone
Michael Worth Calfee
Carlos Henrique Camargo
Andrew Capaldi
Alessandra Carattoli
Ryan B. Carnegie
Giovanna Carpi
Laura M. Carroll
Ronan K. Carroll
Vern B. Carruthers
Josep Casadesús
Santiago Castillo-Ramírez
Clayton C. Caswell
Lindsay J. Caverly
Rodrigo Cayô
Jiri Cerny
Baofeng Chai
Yunrong Chai
Debopam Chakrabarty
C. Chakraborty
Douglas L. Chalker
Gary C. Chan
Josephine R. Chandler
Michael Chandler
Yanjie (Jay) Chao
Dominique Chaput
Sujata S. Chaudhari
Neeraj Chauhan
Damien Chaussabel
Feng Chen
Tingting Chen
Xinhua Chen
Gong Cheng
Jackie K. Cheung
Michaelle Chojnacki
Vincent T. K. Chow
Joseph A. Christie-Oleza
George K. Christophides
Konstantin Chumakov
Richard B. Clark
Christine Clayton
Anna R. Cliffe
Elaine Cloutman-Green
Phillip S. Coburn
Adam S. Cockrell
Carolina Coelho
Alexander M. Cole
Jérôme Collemare
James Collins
Sean Colloms
Teresa M. Coque
Pierre Cornelis
Bruno Correia
Alfred Cortés
Thérèse Couderc
Victoria H. Cowling
Robert A. Cramer
John K. Crane
Max Cravener
Nicholas Crossland
Sean Crosson
Liwang Cui
Paul J. Cullen
Gordon Fritz Custer
Weijun Dai
Ross Edgar Dalbey
Maura Dandri
David J. Davido
Dana A. Davis
Scott C. Dawson
Elizabeth N. De Gaspari
John P. Dekker
Nicholas R. De Lay
Frank R. DeLeo
Emma L. Denham
Charlotte De Rudder
Lalitagauri Deshpande
Rik L. de Swart
Adam M. Deutschbauer
Anthony DeVico
Santosh Dhakal
Vijaykrishna Dhanasekaran
Patricia I. Diaz
Robert P. Dickson
Vincenzo Di Pilato
Eunsoo Do
Ulrich Dobrindt
Tobias Doerr
Yohei Doi
Maria Dominguez-Bello
Justin J. Donato
Tao G. Dong
Anna Dongari-Bagtzoglou
Laurent Dortet
Fernanda Fernandes dos Santos
Zhicheng Dou
Benoît Doublet
Charles M. Dozois
Jan Felix Drexler
Marc Drolet
Milton T. Drott
Kirk Druey
Anamika Dubey
Breck A. Duerkop
Gary M. Dunny
Edison Luiz Durigon
Shanta Dutta
Jeffrey D. Dvorin
Michelle Dziejman
Andrew Edwards
Kathryn M. Edwards
Garth D. Ehrlich
Daniel Elleder
Jeremy R. Ellermeier
Samantha Emma Elizabeth Ellis
Moamen M. Elmassry
Iuliana V. Ene
Melinda Anne Engevik
Seong Kug Eo
Joachim F. Ernst
Alice L. Erwin
Olivier Espeli
Mehrbod Estaki
Prahathees J. Eswara
Christina S. Faherty
John M. Fairbrother
Wenxia Fang
Seamus Fanning
Amir Farnoud
Rubaiyea Farrukee
Lena Faust
Michael J. Federle
Na Fei
Heinz Feldmann
Youzhi Feng
Rachel C. Fernandez
Isabel Fernández Escapa
Astrid Ferrer
Kenneth A. Fields
Agnes Marie Sa Figueiredo
Scott G. Filler
Stefan Finke
Steven E. Finkel
Douglas K. Fischer
Reinhard Fischer
David A. Fitzpatrick
Daniel P. Flaherty
Sheri Floge
Kevin J. Forsberg
Jennifer Foulke-Abel
Elizabeth M. Fozo
Pilar Francino
Kristi L. Frank
Philipp Franken
David N. Fredricks
Megan Freeman
Teresa Frisan
Ryan Frisch
Friedrich Frischknecht
Georg Fritz
Christopher Fröhlich
Simon Frost
Yoichi Furuya
Iwona Gabriel
Attila Gacser
Naren Gajenthra Kumar
Feng Gao
Robert Garcea
Sarahi L. Garcia
Rodolfo Garcia-Contreras
Genevieve Garriss
Beth A. Garvy
Baoxue Ge
Angie Gelli
Nadezhda German
Rohit Ghai
Mahmoud A. Ghannoum
Nicole Gilbert
Sabine Gilch
Steven R. Gill
Peter Gilligan
Robert D. Gilmore
Janet R. Gilsdorf
Nicolas Gisch
Laura Glenndinning
Erin S. Gloag
Matthew Robert Goddard
Richard Goering
Gustavo H. Goldman
Stephen Goldstein
Jonathan Louis Golob
Maria Gómez Brandón
Nardhy Gomez-Lopez
Ben-Qiang Gong
Chengliang Gong
Jesus Gonzalo-Asensio
Uri Gophna
Isabel Gordo
Claire L. Gorrie
Ria Goswami
Yonatan H. Grad
Ian Grainge
Lisa Gralinski
Daniel A. Green
Stefan J. Green
Chris Greening
Trudy H. Grossman
Vesna Grujcic
Marc-Jan Gubbels
Claudia Guldimann
Ozan Gundogdu
Arthur Günzl
Xiaofeng Guo
Ted Hackstadt
Lubomir Hadjiski
Marisa Haenni
Martin W. Hahn
Constantine Haidaris
Anders P. Hakansson
Rebecca Jane Hall
Ruth M. Hall
Luanne Hall-Stoodley
Richard Hamelin
Neal D. Hammer
Kirsten K. Hanson
Nancy D. Hanson
Clare Harding
J. Marie Hardwick
Christopher Harmer
Eva Harris
Jason B. Harris
Erin Harvey
Cynthia Y. He
Michael Hecker
Nagendra R. Hegde
Jacob Heilmann-Clausen
Bernd Heimrich
Jon Heinrichs
Johannes Helder
Richard F. Helm
Yosra A. Helmy
Els Henckaerts
Vincent Hervé
Mark C. Herzberg
Matthias Hess
Steven Higgins
Penelope Higgs
Julian F. Hillyer
Philip Hinchliffe
B. Joseph Hinnebusch
Florian Hladik
Richard L. Hodinka
Sabine Hofmann-Thiel
Jeffrey Hollomon
Corey C. Holt
Kathryn Elizabeth Holt
Yoshikazu Honda-Okubo
Peiying Hong
David C. Hooper
Tomoyuki Hori
Branka Horvat
Guy Houillon
Kai Hu
Qinxue Hu
Sarah Hu
Andrew D. Huang
Laibin Huang
Lili Huang
Weishan Huang
Diego Huet
Wilhelmina Huston
Tu Anh N. Huynh
Jennifer Hyde
Florenzo Iannone
Kensuke Igarashi
Molly A. Ingersoll
Hanne Ingmer
Ronald M. Iorio
Daniel Irimia
Suzanne Lynn Ishaq
Wataru Iwasaki
Angelo Izzo
Mary Ann Jabra-Rizk
William R. Jacobs
Katy Jeannot
Vicki Jeffers
Matthew L. Jenior
Veronica Jimenez
Dong-Yan Jin
Rongsheng Jin
Christian Jobin
Sophia Johler
Jeremiah G. Johnson
Patricia J. Johnson
Timothy J. Johnson
Bradley D. Jones
Jennifer Jones
Howard S. Judelson
Sandra Junglen
Catherine Juste
Vladimir R. Kaberdin
David Kadosh
Björn F. C. Kafsack
Peter C. Kahn
Markus Kainulainen
Maria Kalamvoki
Lindsay Kalan
Markus Kalkum
Said Kamel
Robert W. Kaminski
Beate Kampmann
Shyam Kandel
Manabu Kanno
Stefan Kappe
Nabil Karah
Petros C. Karakousis
Hazma Umut Karakurt
Lisa Karstens
Yuichiro Kashiyama
Tsutomu Katayama
Sophia Kathariou
Souichiro Kato
Carol A. Kauffman
Ghazi Kayali
Hangjun Ke
Brendan Kelly
Ellen Kenchington
Linda J. Kenney
Suliman Khan
Sahil Khanna
Megan R. Kiedrowski
Nicolas Kieffer
Hye Kwon Kim
Jae-Ouk Kim
Lee Kimbell
Janine Kimpel
Gary M. King
Samantha Jane King
Joseph A. Kirk
Lea-Ann S. Kirkham
Laura A. Kirkman
Todd Kitten
Hiroshi Kiyono
Michiel Kleerebezem
Bruce S. Klein
Andreas Klos
Laura J. Knoll
Dennis C. Ko
Wolfgang Koester
Michael H. Kogut
Alain Kohl
Abimbola Kolawole
Nicholas Komar
Heidi Kong
James B. Konopka
Eugene V. Koonin
Ingo Köper
Milena Kordalewska
Omry Koren
Nicole Marie Koropatkin
Natalia Korotkova
Anita A. Koshy
Takahiko Koyama
Lukasz Kozubowski
Florian Krammer
Laurent Kremer
Eric S. Krukonis
Michael D. Kruppa
Jason Kubinak
Kengo Kubota
Andreas Kuhn
Jens H. Kuhn
Eric Kuhnert
Anuj Kumar
Beate Mareike Kümmerer
Harry D. Kurtz
Hiroyuki Kusada
Joseph L. Kuti
Sebla Bulent Kutluay
Jennie H. Kwon
Monique Lafon
Eric R. Lafontaine
Renee M. Laird
Laura Lamb
Tilman Lamparter
Stephanie Langel
Mary L. Lapé-Nixon
Marilynn A. Larson
Christian Lauber
Adam S. Lauring
Catherine Lavazec
Allen Lee
Samuel A. Lee
Soo Chan Lee
Vincent T. Lee
Yong-Hwan Lee
McKenzie K. Lehman
Benfang Lei
Jørgen J. Leisner
Christopher W. Lennon
Dion Lepp
Jhansi L. Leslie
Johan Leveau
Steve Lever
Celine M. Levesque
Zachary A. Lewis
Bin Li
Lin-Xi Li
Min Li
Peng Li
Ziyin Li
Xiao-Ping Liao
Shen Jean Lim
Dominique H. Limoli
Andrew H. Limper
Jonathan Y. Lin
Michael Lin
Nilton Lincopan
Jodi A. Lindsay
Zhuoren Ling
Jacqueline Callihan Linnes
Martin Linster
Peter N. Lipke
Tingli Liu
Zhixia Liu
María A. Llamas
Karen G. Lloyd
Michael K. Lo
Naeemah Logan
Sarah Londrigan
Allison Lopatkin
Jose L. Lopez-Ribot
Alessio Lorusso
Sebastian Lourido
Shirley Luckhart
Ruth Ann Luna
Xin M. Luo
Kalpana Luthra
Joseph Lutkenhaus
Hinh Ly
Michael Lyu
Bing Ma
Li-Jun Ma
Milton Maciel
Erich R. Mackow
Calman A. MacLennan
Eric Mai
Michael H. Malamy
Vera Manageiro
Melissa Manus
Jennifer A. Manuzak
Richard T. Marconi
Rachel Leah Marine
Kevin Maringer
Sara Marti
Emily Toth Martin
Javier Martin
Luis R. Martinez
Jose-Luis Martinez-Gonzalez
Thorsten Mascher
Sébastien Matamoros
Cyrille Mathieu
Jyl S. Matson
Jelle Matthijnssens
Joseph McBride
Shonna M. McBride
Bruce A. McClane
Andrea McCollum
John K. McCormick
Claire E. McCoy
John T. McCrone
Larry S. McDaniel
Diane McDougald
Anita K. McElroy
Noel G. McElvaney
Quinn S. McFrederick
Patrick McGann
Martin J. McGavin
James B. McKinlay
Robert J. McLean
David N. McMurray
Ryan Philip McNamara
Gareth M. McVicker
Anita F. Meier
Olivier Meilhac
Asuncion Mejias
Roberto G. Melano
Jay Mellies
Esther Menendez
Paola E. Mera
Tod J. Merkel
Craig Meyers
Matthew Mikoleit
Laura Milazzo
Andrew Millard
Yves Millemann
Aaron W. Miller
Alita A. Miller
Chad Mire
Yoshiyuki Mishima
Bibhuti Mishra
Nagendra N. Mishra
Satoshi Mitarai
Eliane Namie Miyaji
Kazufumi Mochizuki
Luke A. Moe
Andrew Moeller
Jeffrey Moffit
Maria F. Mojica
Istvan Molnar
Jonathan Monk
David P. Moore
Robert J. Moore
Nathaniel John Moorman
Luís Fernando de Sousa Moraes
Tiago Facury Moreira
Hiroshi Mori
Joachim Morschhäuser
Mary Motyl
Quézia Moura
Paula J. Mouser
Ahmed M. Moustafa
W. Scott Moye-Rowley
Monica Mugnier
Vasant Muralidharan
Isabel Muro-Pastor
Eain A. Murphy
Timothy F. Murphy
Mustapha M. Mustapha
Tin Tin Myaing
Thierry Naas
Anusha Naganathan
Moon H. Nahm
Ryosuke Nakai
Cindy H. Nakatsu
Koji Nakayama
Franz Narberhaus
Farooq Nasar
Luís Cláudio Nascimento da Silva
Sonia Navas-Martin
Erin M. Nawrocki
David M. Needham
Cassandra E. Nelson
Ujjwal Neogi
Jeniel E. Nett
Benjamin W. Neuman
Felipe Piedade Gonçalves Neves
Stephanie L. Neville
Duane W. Newton
Irene L. G. Newton
Cheryl A. Nickerson
Marisa Fabiana Nicolás
Angela M. Nilius
Angela Helen Nobbs
Peter A. Noble
Carolina Silva Nodari
Marc Noguera-Julian
Romolo Nonno
Patrice Nordmann
Steven J. Norris
Jeanette M. Norton
Dominik Nörz
Ângela Novais
Mairi C. Noverr
Tomoyoshi Nozaki
Takuro Nunoura
Brian B. Oakley
Joshua Obar
Shelby L. O’Connor
David O’Dwyer
Peter Oelschlaeger
James P. O’Gara
Naoya Ohara
Shigefumi Okamoto
Yusuke Okazaki
Faten Okda
Andrew J. Olive
Michael Olson
Teresa O’Meara
Troy O’Neil
Eng Eong Ooi
Andres Opazo-Capurro
William D. Orsi
Eric Oswald
George O’Toole
C. Mark Ott
Elizabeth A. Ottesen
Marc Ouellette
Mark S. Paget
Tanapat Palaga
Raghavan U. Palaniappan
Mitchell Pallett
Satheshkumar Panayampalli
John C. Panepinto
Costas C. Papagiannitsis
Daniel Paredes-Sabja
Joanna L. Parish
Dan Mcfarland Park
Hee-Soo Park
Jason Park
Dane Parker
Donovan H. Parks
Colin R. Parrish
Christopher M. Parry
Sally R. Partridge
Erica Pasini
David Paterson
Timothy C. Paulitz
Matthew Pauly
Jovan Pavlovic
Helene M. M. Paxton
Andrew Pekosz
Xinxia Peng
Jose Christian Perez
Benjamin Perin
Andreas Peschel
Brian Peters
Felicitas Pfeifer
Mrudula Phadke
Birgit Piechulla
Andrzej Piekarowicz
Shmuel Pietrokovski
Tatiana C. A. Pinto
Johann Pitout
Gregory V. Plano
Randall Platt
Daniel Pletzer
Carolina H. Pohl
Laurent Poirel
Christopher Robert Polage
Shawn W. Polson
Frederic Poly
María Teresa Ponce-Noyola
David L. Popham
Erin P. Price
Sean T. Prigge
Harry E. Prince
Peter M. Pryciak
Michael J. Pucci
Alexandra E. Purdy
Nicole E. Putnam
Dohun Pyeon
Lizeng Qin
Jianming Qiu
Pilar Quintana
Robert G. Quivey
Brent Race
Govindarajan Rajagopalan
Michael Rak
Srinivasan Ramakrishnan
Kumaran S. Ramamurthi
Christophe Ramière
Maria Soledad Ramirez
Cayo Ramos
Chad A. Rappleye
David Rasko
Adam J. Ratner
R. S. Redman
Peter Reeves
Barbara Rehermann
Aaron Reinke
Lina Reslan
Peter Reuther
Matthew R. Reynolds
Todd B. Reynolds
Jonathan Richards
Bert K. Rima
Marilyn C. Roberts
Derrick R. Robinson
Marcio L. Rodrigues
Estefania Rodriguez
Geraint B. Rogers
Robin Rebecca Rohwer
Mohsen Rokni
Sandra Romero-Steiner
R. Martin Roop
Alexandre Soares Rosado
Jason W. Rosch
Jean-Pierre Routy
Emeline Roux
Craig R. Roy
Nicole Roy
Polly Roy
Alex Rubinsteyn
Christian J. Rudolph
Cristian Ruiz
Olena Rzhepishevska
Wilmara Salgado-Pabón
Amali Samarasinghe
Linus Sandegren
Felipe H. Santiago-Tirado
Ezequiel Santillan
Clarissa Santos Rocha
Toyotaka Sato
Charles A. Scanga
Joy Scaria
Jeffrey W. Schertzer
Patrick D. Schloss
Eric Schmidt
Jennifer Elise Schmidt
Thomas Mitchell Schmidt
Volker Schmidt
Mirco Schmolke
Tony Schountz
Michael Schuit
Christian Schwerk
Jarrod J. Scott
Amin Sedokani
Anna Maria Seekatz
Julie A. Segre
Kate L. Seib
H. Steven Seifert
Rangaraj Selvarangan
Hidenobu Senpuku
Keun Seok Seo
Aswin Sai Narain Seshasayee
William M. Shafer
Dilip Shah
Priya S. Shah
Shiraz A. Shah
Kathy Ho Yen Shair
Dipali Sharma
Jessica R. Sheldon
Aimee Shen
Bang Shen
Mang Shi
Shinsuke Shigeto
Shin-Ru Shih
Teppei Shimiasaki
Daisuke Shiomi
Nahum Y. Shpigel
Joshua D. Shrout
Shahid Siddique
Anita Sil
Roberto Silva
Lynn L. Silver
Monique Simier
Anthony P. Sinai
Steven M. Singer
Upinder Singh
Albert Siryaporn
Jerod A. Skyberg
Renata Dezengrini Slhessarenko
Monika Słomińska-Wojewódzka
Carolyn M. Slupsky
Mark S. Smeltzer
Darian Smercina
Darci Smith
James Leif Smith
Joseph D. Smith
Wiep Klaas Smits
Teemu Smura
Evan S. Snitkin
Jonathan W. Snow
Milena Sokolowska
Evgeni Sokurenko
David R. Soll
Harini Sooryanarain
Joseph A. Sorg
Daniel Stadlbauer
Renske D. M. Steenbergen
Lisa Y. Stein
Eike Steinmann
David Cole Stevens
Dennis Stevens
Brian Stevenson
David B. Stewart
Scott Stibitz
Ashley L. St. John
Gregory G. Stone
Daniel Straume
Frank Stubenrauch
Karina Stucken
Jorg Stulke
Carlos S. Subauste
David Sue
Paul M. Sullam
Jie Sun
Shan Sun
Maarit Suomalainen
Ivan Surovtsev
Michael Surrette
Joyce Sutcliffe
Mehul S. Suthar
Troy C. Sutton
Elena Suvorova
Staffan G. Svärd
Maxim S. Svetlov
Joel Swanson
W. Edward Swords
Francois-Etienne Sylvain
Rita Tamayo
Ming Tan
Benjamin Tang
Yuyang Tang
Arnaud Taton
Miguel Cacho Teixeira
Akihiko Terada
Benno Herman Ter Kuile
Ken Teter
Rajani Thanissery
Kevin R. Theis
Casey M. Theriot
Gavin H. Thomas
Elizabeth Thrall
Thomas Thurnheer
Andrea Ticinesi
Rafal Tokarz
Andrew Tomaras
Steven Y. C. Tong
Olivera Topalović
Tadashi Toyama
Stephen Tristram
Nicolas Tromas
François Trottein
Takafumi Tsuboi
Claire Elizabeth Turner
Kenneth L. Tyler
Gregory Tyrrell
Juan Esteban Ugalde
David Ulaeto
Imran Ullah
Gottfried Unden
Priya Uppuluri
Syun-Ichi Urayama
Olivier Vallon
Miguel A. Valvano
Chris Van Beneden
Kurt Jason Vandegrift
Dan Vanderpool
Adrianus W. M. van der Velden
Patrick Van Dijck
Mark W. J. van Passel
Debby van Riel
Vicky L. van Santen
Brian D. VanScoy
Daria Van Tyne
Arvind Varsani
Joel Vega-Rodríguez
Lonneke Vervelde
Alejandro J. Vila
Vladimir Vinnik
Joerg Vogel
Chantal B. Vogels
Robin Voigt-Zuwala
Petr Volf
Veronika von Messling
Jay Vornhagen
Daniel E. Voth
Jo-Marie Vreulink
Jatin M. Vyas
Joseph Thomas Wade
Rezwanul Wahid
Matthew K. Waldor
Seth T. Walk
Louise A. Walker
Mark J. Walker
Daniel Wall
Nicholas A. Wallace
Judd Walson
Jens Walter
Hui Wang
Jincheng Wang
Joyce Wang
Jue D. Wang
Xiaoxue Wang
Yan Wang
Yang Wang
Jonathan Mark Warawa
Matthew J. Wargo
Christopher M. Waters
Nadeeka Kumari Wawegama
Keith E. Weaver
Richard J. Webby
Xin Wei
Brian C. Weinrick
Louis M. Weiss
Eric Wenzler
Dawn Wetzel
Nicole Wheeler
Stephen C. Whisson
Gregory Whitaker
Jason K. Whitmire
Gottfried Wilharm
Julia Willett
Emma H. Wilson
Richard A. Wilson
Steven S. Witkin
Christiane E. Wobus
Alan J. Wolfe
Michael H. Woodworth
Karen Wozniak
Rachel A. F. Wozniak
Gerard D. Wright
Nicholas C. Wu
William H. Wunner
Susan Wyllie
Kelly Leanne Wyres
Han Xia
Hang Xie
Jin-Rong Xu
Xiyuan Xu
Yuquan Xu
Chaoyang Xue
Timothy L. Yahr
S. Steve Yan
Hee-Jeong Yang
Yang Yang
Ye Yang
Zhaomin Yang
Catherine Yoshida
Yang Yu
Yunsong Yu
Min Yue
Joseph P. Zackular
Rahat Zaheer
Kamarul Zarkasi
Raffaele Zarrilli
Michael Zasloff
Anna C. Zemke
Gabriel E. Zentner
Bing Zhai
Yuanchao Zhan
Cheng-Cai Zhang
Chiyu Zhang
Rui Zhang
Jiangchao Zhao
Beiwen Zheng
Hao Zheng
Guangming Zhong
Xiaohui Zhou
Ran Zichel
Lisa Ziegler
Seth Zost
Abdelrahman Zueter
